# Distinct age-related effects of homologous recombination deficiency on genomic profiling and treatment efficacy in gastric cancer

**DOI:** 10.1007/s00535-025-02267-3

**Published:** 2025-06-13

**Authors:** Yoshie Maki, Yoshiyasu Kono, Toshiki Ozato, Hideki Yamamoto, Akira Hirasawa, Daisuke Ennishi, Shuta Tomida, Shinichi Toyooka, Kenta Hamada, Masaya Iwamuro, Seiji Kawano, Motoyuki Otsuka

**Affiliations:** 1 Faculty of Medicine, Department of Gastroenterology and Hepatology, Dentistry and Pharmaceutical Sciences, Okayama University, 2-5-1 Shikata-Cho, Kita-Ku, Okayama 700-8558 Japan; 2 Department of Clinical Genomic Medicine, Okayama University Hospital, 2-5-1 Shikata-Cho, Kita-Ku, Okayama 700-8558 Japan; 3 Center for Comprehensive Genomic Medicine, Okayama University Hospital, 2-5-1 Shikata-Cho, Kita-Ku, Okayama 700-8558 Japan; 4 Faculty of Medicine, Department of General Thoracic Surgery, Breast and Endocrinological Surgery, Dentistry and Pharmaceutical Sciences, Okayama University, 2-5-1 Shikata-Cho, Kitaku, Okayama 700-8558 Japan; 5 Faculty of Medicine, Department of Practical Gastrointestinal Endoscopy, Dentistry and Pharmaceutical Sciences, Okayama University, 2-5-1 Shikata-Cho, Kitaku, Okayama 700-8558 Japan; 6 Department of Gastroenterology, Okayama University Hospital, 2-5-1 Shikata-Cho, Kita-Ku, Okayama 700-8558 Japan

**Keywords:** Homologous recombination repair gene, Early-onset gastric cancer, Comprehensive genomic profiling

## Abstract

**Background:**

The incidence of gastric cancer among younger patients is increasing globally, with growing attention being paid to the role of homologous recombination deficiency (HRD). However, the effect of HRD on treatment outcomes and prognosis in this population remains unclear.

**Methods:**

We analyzed clinical and genomic data from the Center for Cancer Genomics and Advanced Therapeutics database. Younger patients (≤ 39 years, *n* = 140) were compared with older patients (≥ 65 years, *n* = 1118) diagnosed with gastric cancer. This study focused on mutations in homologous recombination repair (HRR) genes and their association with tumor mutation burden (TMB), microsatellite instability (MSI), and treatment outcomes.

**Results:**

In older patients, HRD was associated with higher TMB and microsatellite instability-high (MSI-H) status, whereas no such correlations were observed in younger patients. Notably, MSI-H status was not observed in the younger group. Younger patients with HRD had a significantly shorter time to treatment failure (TTF) and overall survival (OS) than those without HRD. Conversely, in older patients, there was no significant difference in TTF or OS based on HRD status.

**Conclusion:**

HRR gene mutations influence genomic profiling, TMB, and MSI differently depending on the age of gastric cancer onset, suggesting potential effects on treatment efficacy and prognosis.

## Introduction

The incidence of gastric cancer among adolescents and young adults has been increasing globally, with younger patients generally having poorer prognoses than middle-aged and older adults [1, 2]. Despite this trend, comprehensive genomic profiling and clinical characteristics of gastric cancer in younger patients remain poorly understood.

Homologous recombination deficiency (HRD) has recently emerged as a key factor in the pathogenesis of various cancers, including gastric cancer. A recent study linked germline HRD and *Helicobacter pylori* infection to early-onset gastric cancer [3], while most studies on early-onset gastric cancer have focused primarily on germline mutations [3–5], and there is a growing need to examine comprehensive variants, not limited to germline mutations, to understand the genetic landscape of this disease fully.

HRD has been associated with a high tumor mutation burden (TMB), which is thought to enhance the efficacy of immune checkpoint inhibitors [6, 7]. Additionally, HRD has been shown to improve the effectiveness of platinum-based chemotherapy, particularly in gynecological cancers [8, 9]. However, the influence of HRD and TMB on the treatment efficacy and prognosis of gastric cancer, especially with respect to the age of onset, remains understudied.

In this study, we assessed the effect of HRD on treatment efficacy and prognosis in younger and older patients with GC, using a large-scale national genomic profiling cohort. Our findings reveal distinct age-related differences. The influence of HRD on TMB and microsatellite instability (MSI) varies significantly with age, contributing to differences in treatment response and prognosis.

## Methods

### Study design and population

We conducted a retrospective observational study using the clinical and genomic data of patients with advanced or recurrent gastric cancer registered at the Center for Cancer Genomics and Advanced Therapeutics (C-CAT) utilization portal between June 2019 and December 2023. Data were extracted from two cohorts based on age at the time of C-CAT registration: a younger group (age ≤ 39 years; n = 140) and an older group (age ≥ 65 years; *n* = 1118). This study complied with the principles outlined in the Declaration of Helsinki. It was approved by our institutional Ethics Committee (approval number: 2111-047) as well as the C-CAT review board (C-CAT Control Number: CDU2022-012E02).

### Data collection and annotation

Clinical information, including patient characteristics and treatment-related details, such as age, sex, primary tumor site, predominant histological type, Eastern Cooperative Oncology Group Performance Status (ECOG PS), family history of cancer, smoking and drinking habits, human epidermal growth factor receptor 2 (HER2) status, type of comprehensive genomic profiling (CGP) test, tissue sampling site, and chemotherapy regimens with treatment lines, was collected. We analyzed five key predisposing genes for gastric cancer (*TP53*, *KRAS*, *APC*, *SMAD4*, and *CDH1*), 16 homologous recombination repair (HRR) genes (*ARID1A, ATM, ATRX, BAP1, BARD1, BRCA1, BRCA2, BRIP1, CDK12, FANCA, FANCC, MRE11, PALB2, PTEN, RAD51D*, and *RAD51C*), and mismatch repair (MMR) genes (*MSH6*, *MLH1, MSH2*, and *PMS2*), as well as MSI status and TMB. To ensure relevance, only genetic variants classified as 'oncogenic,' 'pathogenic,' 'likely oncogenic,' or 'likely pathogenic' based on C-CAT clinical interpretation were included, while variants of unknown significance were excluded. Clinical annotation using C-CAT was based on the Cancer Knowledge Database, which compiles information on gene mutations, drugs, and clinical trials from global genomic medicine databases [10].

### Comprehensive genomic profiling assays and quality control

Several CGP assays are currently being used in clinical practice. Foundation One CDx (Foundation Medicine), OncoGuide™ NCC Oncopanel System (Sysmex Corporation), and GenMine TOP (Konica Minolta Inc.) were used to analyze formalin-fixed paraffin-embedded (FFPE) tumor tissue samples. Foundation One Liquid CDx (Foundation Medicine) and Guardant 360 CDx (Guardant Health Incorporated) were used for circulating tumor DNA analysis. Quality control (QC) assessments of nucleic acids, including checks for yield, concentration, and fragmentation, were conducted before submitting the samples for panel testing. Tissue panel testing was performed if QC results met the required standards. Liquid-based panel testing is performed when quality issues or low nucleic acid yields are encountered.

### Comparative analysis of genomic features and survival outcomes

We first compared the features of CGP, with a particular focus on HRR genes, between the younger (*n* = 140) and older (*n* = 1118) groups. To visualize the differences in the genomic landscape between the two groups, we generated an Oncoprint that included genomic alteration data, such as single-nucleotide variants (SNVs), copy number variants (CNVs), MSI, and TMB, as well as clinicopathological information, including sex, primary tumor location, and histology. Subsequently, we evaluated the frequency of HRR gene variants in the two groups, and the relationship between HRR gene variants, MSI status, and TMB.

Next, we examined the effect of HRD on the survival and therapeutic efficacy of platinum-based doublet chemotherapy as the first-line regimen in both groups. Overall survival (OS) and time to treatment failure (TTF) were also analyzed. Overall survival (OS) was defined as the interval between the start of treatment and death. TTF was defined as the duration from the initiation of treatment to either the discontinuation of therapy or death from any cause. HRD was defined as the presence of one or more variants in HRR genes following previous reports [11, 12]. For OS analysis, patients enrolled in clinical trials and those lacking final survival or follow-up data were excluded. For the TTF analysis, only patients who received platinum-based doublet chemotherapy as the first-line regimen were included. Patients enrolled in the clinical trials, those with missing data, and those who received neoadjuvant or postoperative chemotherapy were excluded from the study.

### Statistical analysis

Categorical variables were compared using chi-square tests, and continuous variables were evaluated using Student’s *t* test. OS and TTF were estimated with 95% confidence intervals (CI) using the Kaplan–Meier method and compared between groups using the log-rank test. All statistical analyses were two-tailed, and a *P* value of less than 0.05 was considered to indicate statistical significance. Statistical computations were performed using JMP Pro 17 software (SAS Institute Inc., Cary, NC, USA).

## Results

### Patient characteristics

**Fig. 1:**
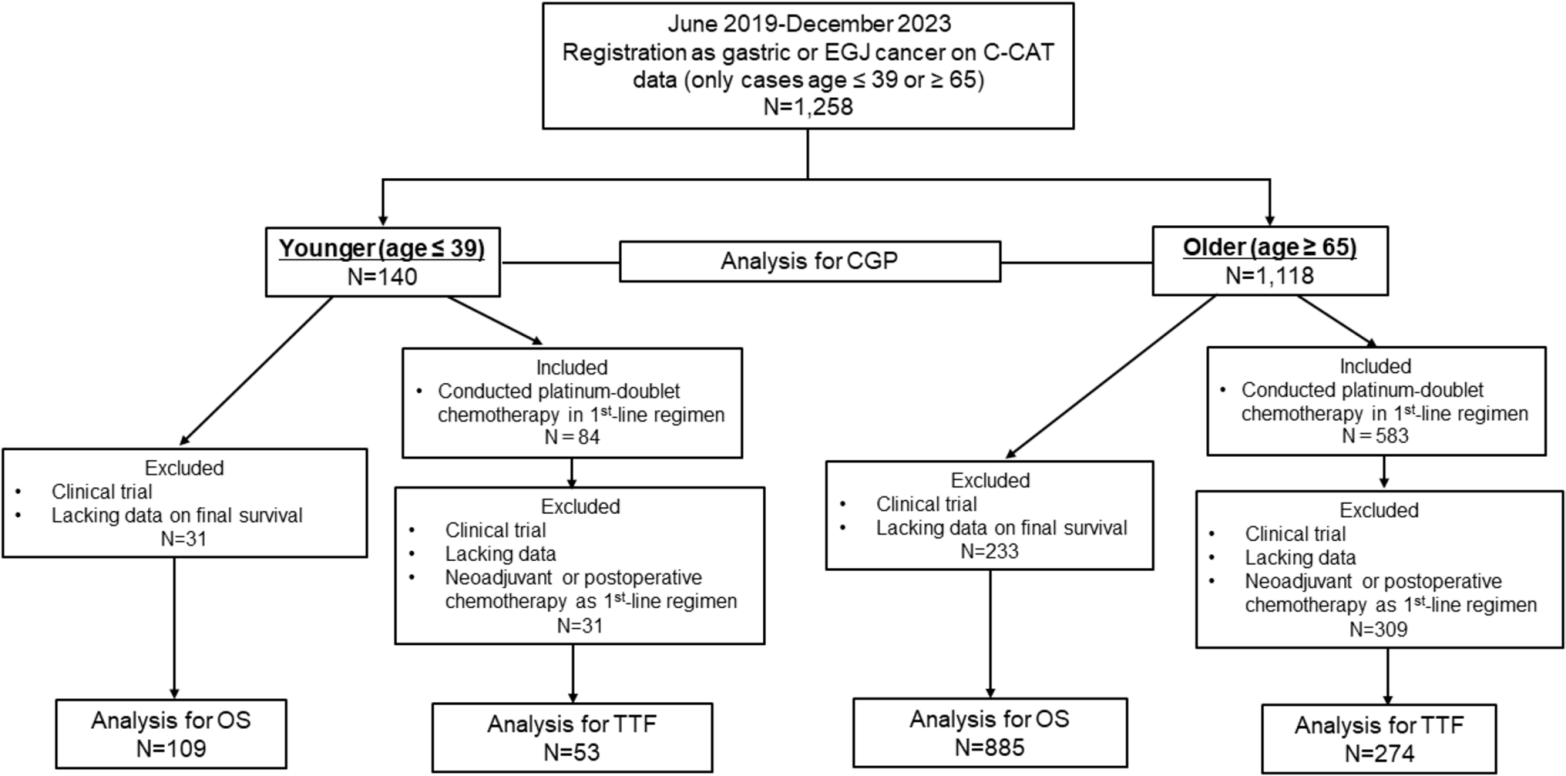
Study flowchart. We identified 1,258 patients with gastric or esophagogastric junction cancer from the Center for Cancer Genomics and Advanced Therapeutics database between June 2019 and December 2023, divided into a younger group (≤ 39 years; *n* = 140) and an older group (≥ 65 years; *n* = 1118). Comprehensive genomic profiling test was performed in all patients. Overall survival (OS) and time to treatment failure (TTF) were analyzed in patients receiving first-line platinum-doublet chemotherapy, excluding those in clinical trials or with missing data. Consequently, 109 and 885 patients in the younger and older groups, respectively, were included in OS analysis. Similarly, 53 and 274 patients in the younger and older groups, respectively, were included in the TTF analysis. *N* number, *EGJ* esophagogastric junction, *C-CAT* Center for Cancer Genomics and Advanced Therapeutics, *CGP* comprehensive genomic profiling, *OS* overall survival, *TTF* time to treatment failure

This study included 1258 patients with advanced or recurrent gastric or EGJ cancer, divided into 140 in the younger group (age ≤ 39 years) and 1118 in the older group (age ≥ 65 years). CGP analysis was performed on all patients. OS was assessed in 109 patients from the younger group and 885 patients from the older group. In contrast, TTF for platinum-based chemotherapy was analyzed in 53 younger and 274 older patients after excluding ineligible cases, as shown in the eligibility flowchart (Fig. 1).

Table 1 illustrates the clinical characteristics of the enrolled patients, including sex, primary tumor site, predominant histological type, ECOG PS, family history of cancer, smoking and alcohol consumption habits, HER2 score, type of CGP test, and tissue sampling site. The younger group had a significantly higher proportion of female patients (*P* < 0.001). The diffuse type was more prevalent in the younger group, whereas the intestinal type was more frequently observed in the older group (*P* < 0.001). Furthermore, the older group had more smoking (*P* < 0.001) and drinking habits (*P* = 0.0014). Significant differences were also noted in the tissue sampling areas between the two groups (*P* = 0.0002). No significant differences were observed among other factors (Table 1). These demographic and clinical differences highlight the distinct biological and environmental factors that may underlie age-related heterogeneity in gastric cancer.

**Table 1:** Clinical characteristics

	Younger *n* = 140	Older *n* = 1118	*P* value
Sex, *n* (male/female)	64/76	819/299	< 0.001
*Primary tumor site*, *n* (%)			0.53
EGJ	15 (11)	100 (8.9)	
Stomach	125 (89)	1018 (91)	
*Predominant histological type*, *n* (%)			< 0.001
Diffuse	65 (46)	278 (25)	
Intestinal	73 (52)	835 (74)	
Special	2 (1.4)	5 (0.45)	
ECOG PS (0/1/2/3/4/unknown), *n*	83/46/6/0/0/2	546/484/38/6/2/10	0.18
Family history of cancer *n* (%)	101 (74)	742 (68)	0.28
Smoking habit, *n* (%)	33 (24)	648 (60)	< 0.001
Drinking habit, *n* (%)	9 (7.0)	189 (18)	0.0014
HER2 score, *n* (0/1 + /2 + /3 + /unknown)	81/21/7/19/7	535/174/132/132/102	0.060
*Type of comprehensive genomic profiling test*, *n* (%)			0.48
F1CDx	107 (76)	849 (76)	
NOP	18 (13)	110 (9.8)	
F1L	15 (11)	144 (13)	
Guardant 360	0	8 (0.72)	
TOP	0	7 (0.63)	
*Tissue sampling site*, *n* (%)			0.0002
Primary	92 (74)	840 (87)	
Metastatic	33 (26)	126 (13)	

*ECOG PS* Eastern Cooperative Oncology Group performance status, *EGJ* esophagogastric junction, *HER2* human epidermal growth factor receptor 2, *F1CDx* Foundation One CDx, *NOP* OncoGuide NCC Oncopanel System, *F1L* Foundation One Liquid CDx, *Guardant 360* Guardant 360 CDx, *TOP* GenMine TOP, *n* number

### Details of chemotherapy regimens after first-line platinum-doublet chemotherapy

Table 2 presents the details of the regimens administered after the use of platinum-doublet chemotherapy as the first-line treatment. A total of 84 (60%) and 583 (52%) patients in the younger and older groups, respectively, received a platinum-doublet regimen as first-line chemotherapy. Among these patients, the proportion who received taxane-based regimens (with ramucirumab), the standard second-line treatment, did not significantly differ between the groups (younger: 50% vs. older: 58%; *P* = 0.20). In contrast, a significantly higher proportion of younger patients received immune checkpoint inhibitors (ICIs) as part of their first-line platinum-doublet regimen group (32% vs. 15%; *P* = 0.001). Conversely, a significantly higher proportion of older patients received ICIs in the second-line or later setting (56% vs. 21%; *P* < 0.0001). Overall, a significantly higher total proportion of older patients were treated with ICIs during any line of therapy (69% vs. 54%; *P* = 0.0061). The observed differences in ICI use may be influenced by clinical factors such as biomarker expression, treatment accessibility, or physician preference across age groups.

**Table 2:** Details of chemotherapy regimens beyond platinum-doublet chemotherapy in first-line, including second-line regimen (taxane and ramucirumab) and immune checkpoint inhibitors

	Younger *n* = 84	Older *n* = 583	*P* value
Taxane (plus ramucirumab) in 2nd-line regimen	42 (50)	336 (58)	0.20
*Administration of ICIs*, *n* (%)			
First-line (with platinum-doublet)	27 (32)	79 (14)	0.0001
Second-line or later	18 (21)	324 (56)	< 0.0001
Total	45 (54)	403 (69)	0.0061

*ICI* immune checkpoint inhibitor, *n* number

### Genomic profiling and age-related differences

**Fig. 2:**
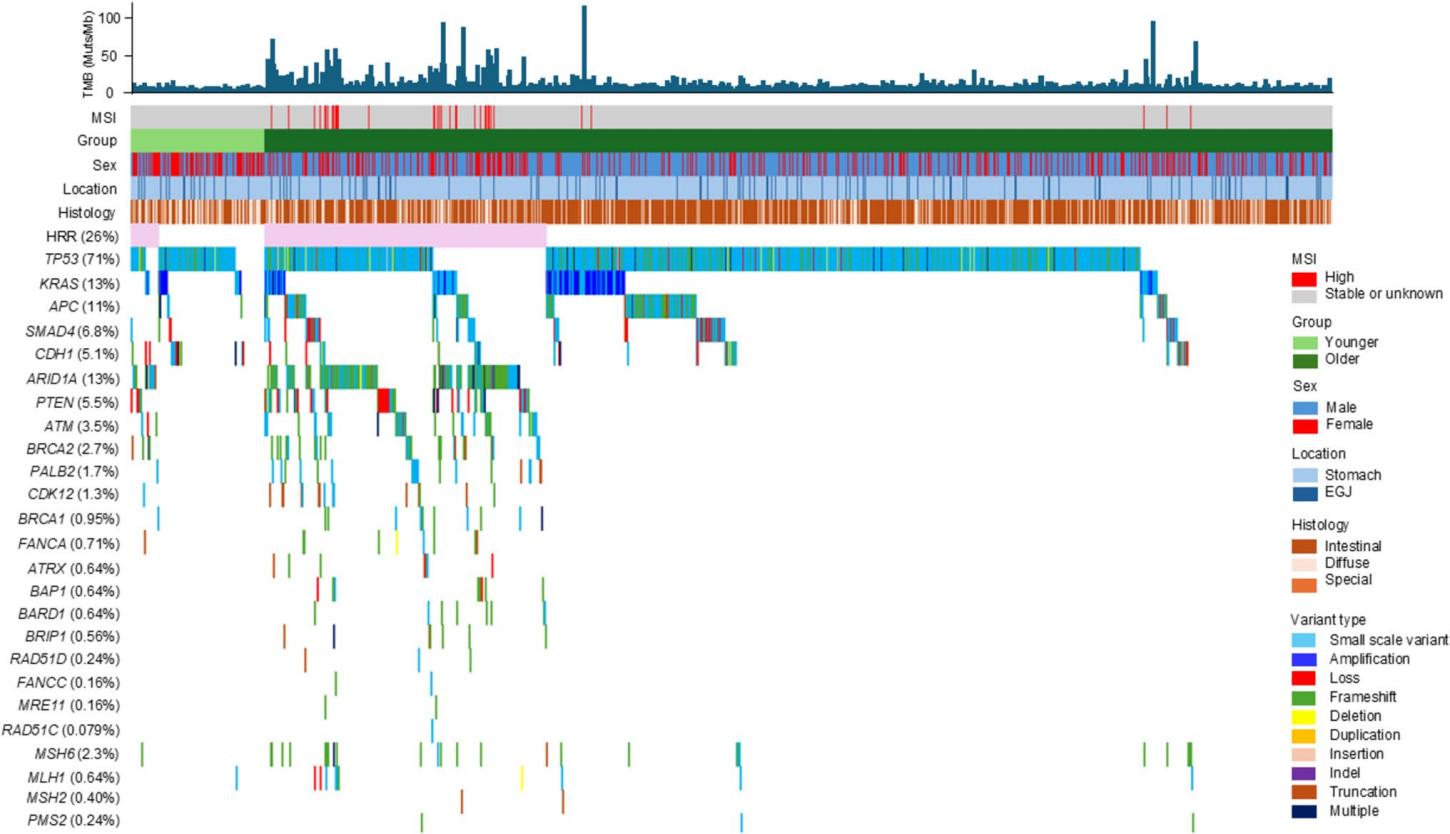
Mutation landscape of 1258 patients. An oncogene was generated to display 16 homologous recombination repair genes (*ARID1A, ATM, ATRX, BAP1, BARD1, BRCA1, BRCA2, BRIP1, CDK12, FANCA, FANCC, MRE11, PALB2, PTEN, RAD51D, and RAD51C*), along with five important predisposing genes for gastric cancer (*TP53, KRAS, APC*, *SMAD4*, and *CDH1*), mismatch repair genes (*MSH6*, *MLH1, MSH2*, and *PMS2*), microsatellite instability, and tumor mutation burden. Patients were categorized into two groups: the younger group (*n* = 140), represented by a light green bar, and the older group (*n* = 1118), represented by a dark green bar. Clinical and molecular features were displayed alongside genetic alterations. *MSI* microsatellite instability, *TMB* tumor mutation burden, *HRR* homologous recombination repair, *EGJ* esophagogastric junction

The differences in genomic profiles between the younger and older groups are illustrated as oncoprints with a particular focus on the HRR genes (Fig. 2). The analysis also included key predisposition genes for gastric cancer (*TP53*, *KRAS*, *APC*, *SMAD4*, and *CDH1*) and MMR genes (*MSH6*, *MLH1*, *MSH2*, and *PMS2*). Additionally, the figure presents data on MSI status, TMB, sex, histological type, and primary tumor site. Notably, no MSI-high (MSI-H) cases were observed in the younger group, and TMB values were lower than those in the older group. *TP53* variants were identified in 71% of the patients. Variants in *CDH1*, a gene associated with hereditary gastric cancer, were found in only 5.1% of the cases. Among the HRR genes, *ARID1A* showed the highest variant frequency (13%), followed by *PTEN* (5.5%) and *ATM* (3.5%). *MSH6* was the most frequently mutated MMR gene, with a variant frequency of 2.3%.

**Fig. 3:**
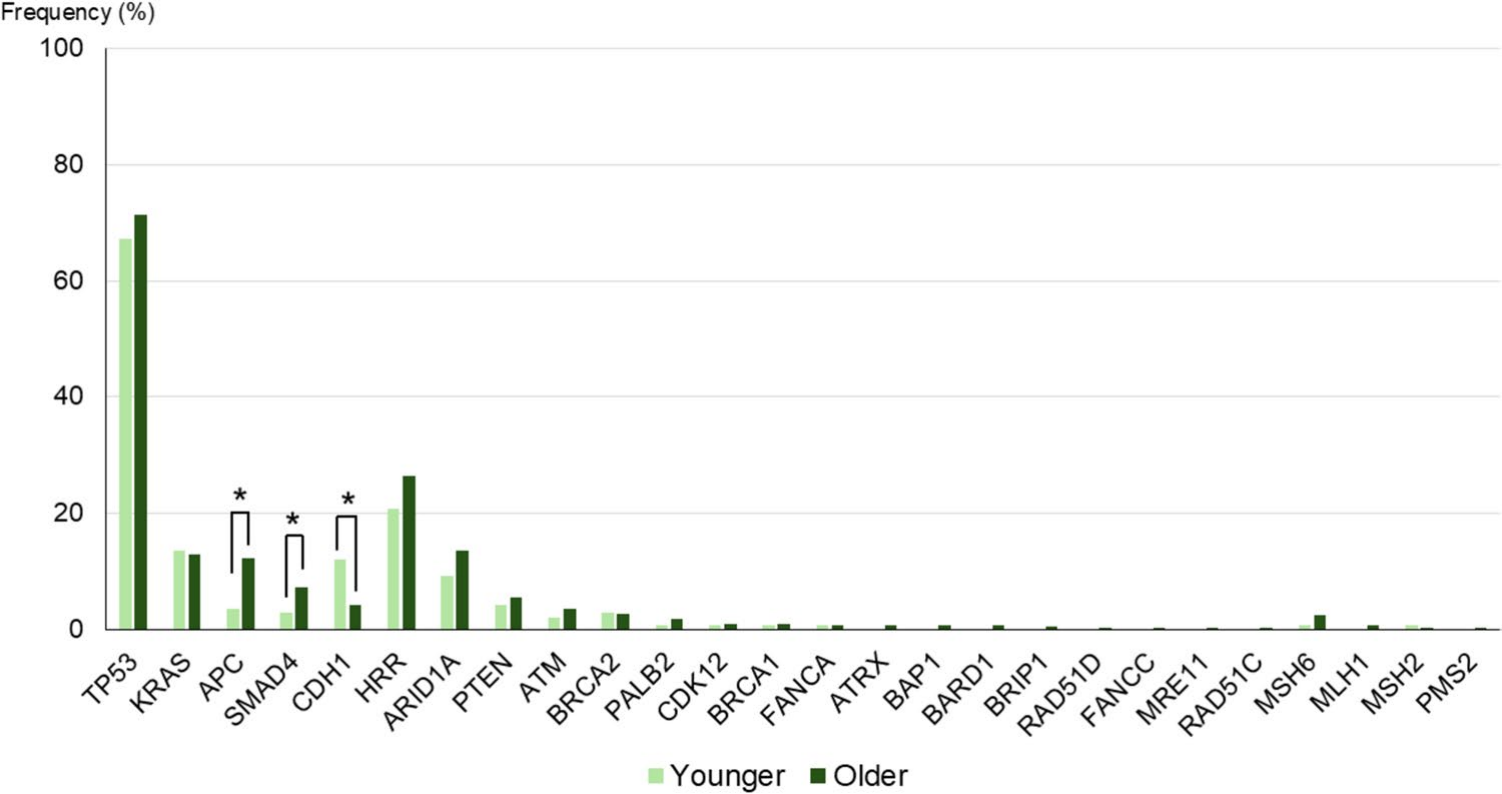
Frequency of each gene variant, including details of homologous recombination repair genes by age group. The bar chart illustrates the variant frequency of each gene, including homologous recombination repair genes. The older group had significantly higher *APC* and *SMAD4* variant frequencies, while *CDH1* variants were more frequent in the younger group. *HRR* homologous recombination repair. *P* < 0.05

Next, we examined the variant frequencies of genes, including HRR, between younger and older groups. There was no significant difference in the frequency of *TP53* variants between the two groups (67% in the younger group vs 71% in the older group, *P* = 0.32). The frequency of *APC* variants was significantly higher in the older group than in the younger group (12% vs 3.6%, *P* < 0.05). Similarly, the frequency of *SMAD4* variants was significantly higher in the older group than in the younger group (7.2% vs 2.9%; *P* < 0.05). In contrast, the frequency of *CDH1* variants was significantly higher in the younger group than in the older group (12% vs 4.2%; *P* < 0.05). The frequency of *ARID1A* variants was higher in the older group than in the younger group although the difference was not statistically significant (14% vs 9.3%, *P* = 0.18). Similarly, although the frequencies of other HRR gene variants were higher in the older group than in the younger group, the differences were not statistically significant. There were no significant differences in the frequencies of MMR genes between the two groups (Fig. 3). These findings indicate subtle but potentially meaningful differences in the mutational landscape of gastric cancer between age groups.

### Age-dependent effects of HRD on MSI status and TMB in gastric cancer

**Fig. 4:**
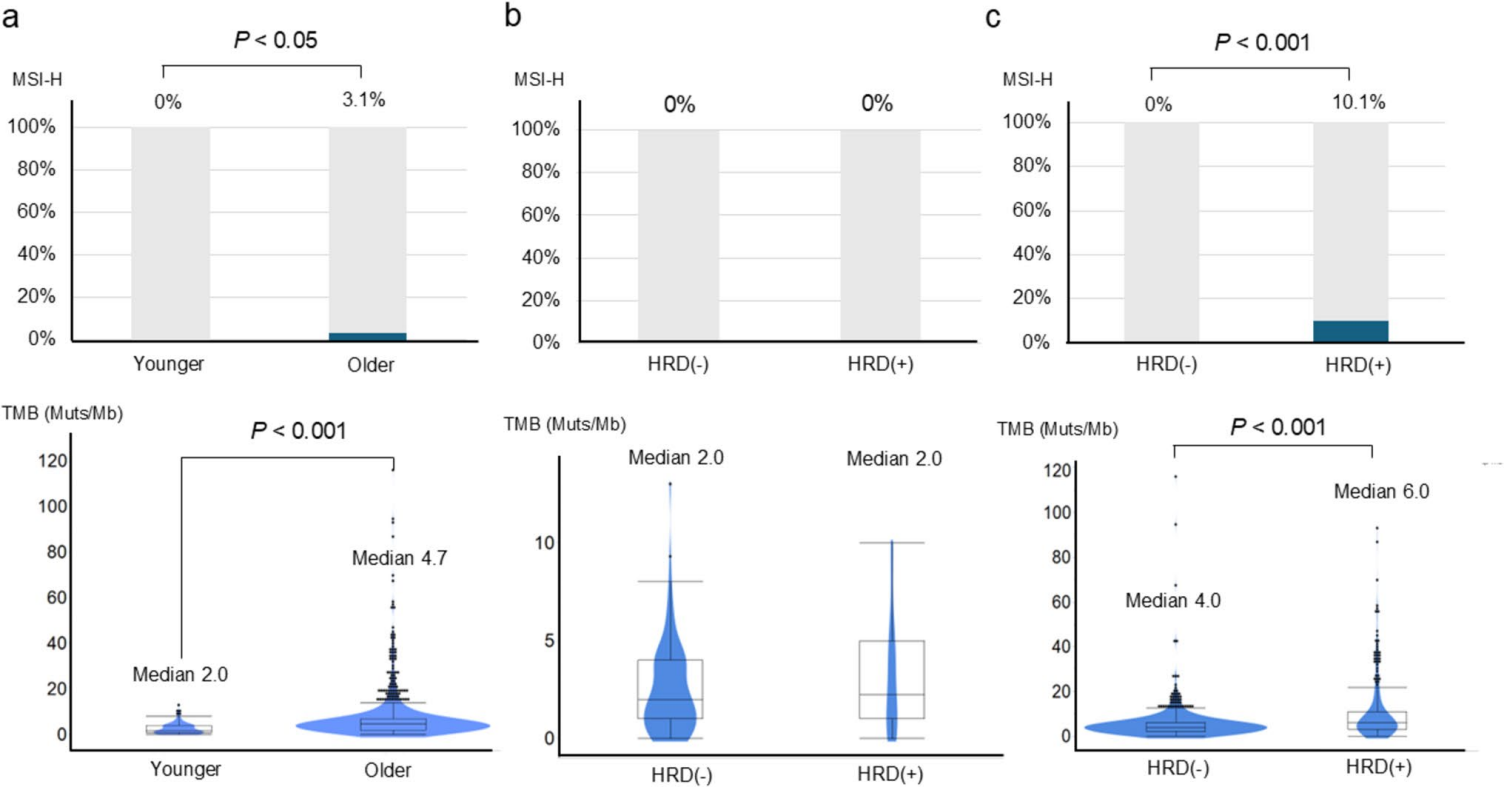
Frequency of microsatellite instability-high and tumor mutation burden values according to age and homologous recombination deficiency status. **a** The frequency of microsatellite instability (MSI-H) was significantly higher in the older group than in the younger group (3.1% vs 0%, *P* < 0.05). Additionally, the median tumor mutation burden (TMB) was significantly higher in the older group than in the younger group (4.7 Muts/Mb vs 2.0 Muts/Mb, *P* < 0.001). **b** No MSI-H cases were observed in either homologous recombination deficiency (HRD)-positive or HRD-negative cases in the younger group. Similarly, the median TMB values were comparable between HRD-positive and HRD-negative cases in this group (2.0 Muts/Mb vs 2.0 Muts/Mb, *P* = 0.49). **c** The frequency of MSI-H was significantly higher in the HRD-positive cases than in the HRD-negative cases in the older group (10.1% vs 0%, *P* < 0.001). Similarly, the median TMB values were significantly higher in HRD-positive cases than in HRD-negative cases in this group (4.0 Muts/Mb vs 6.0 Muts/Mb, *P* < 0.001). *MSI-H* microsatellite instability-high, *TMB* tumor mutation burden, *HRD* homologous recombination deficiency

We assessed the impact of HRD on MSI status and TMB in younger and older groups. The incidence of MSI-H was significantly higher in the older group than in the younger group (3.1% vs 0%, *P* < 0.05). Similarly, the median TMB values were significantly higher in the older group compared to the younger group (4.7 Muts/Mb vs 2.0 Muts/Mb, *P* < 0.001) (Fig. 4a). Notably, no cases of MSI-H were observed in the younger group, regardless of the HRD status. Additionally, the median TMB values were comparable between HRD-positive and HRD-negative cases in the younger group (2.0 Muts/Mb vs 2.0 Muts/Mb, *P* = 0.49) (Fig. 4b). In contrast, the frequency of MSI-H was significantly higher in HRD-positive cases than in HRD-negative cases in the older age groups (10.1% vs 0%, *P* < 0.001). Moreover, the median TMB values were significantly higher in HRD-positive cases compared to HRD-negative cases in the older group (6.0 Muts/Mb vs 4.0 Muts/Mb, *P* < 0.001) (Fig. 4c). These findings suggest that HRD may have distinct effects on MSI status and TMB depending on the age at the onset of gastric cancer.

### Prognostic impact of HRD on platinum-doublet regimens in younger and older gastric cancer patients

**Fig. 5:**
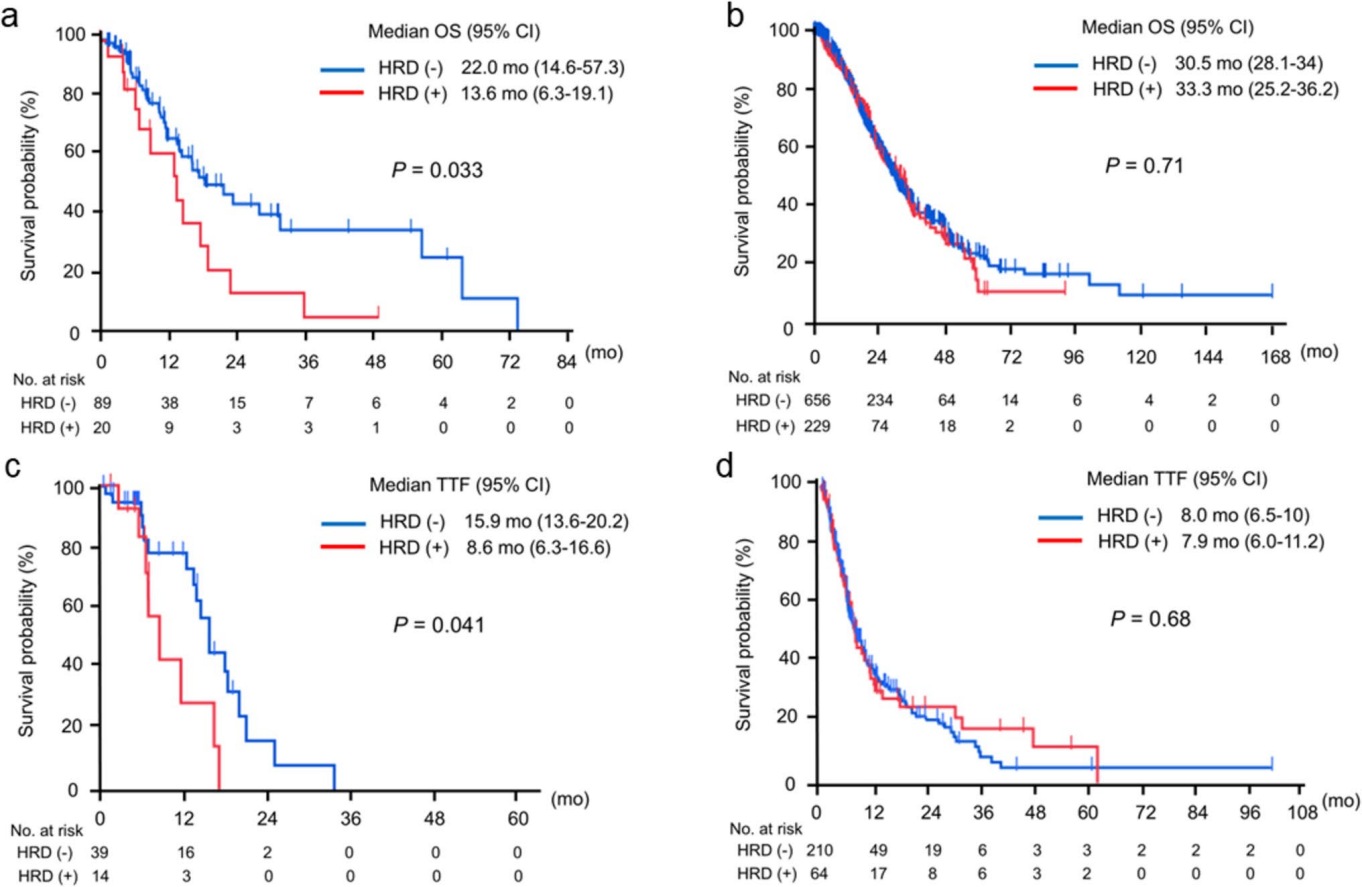
Overall survival and time to treatment failure for the platinum-doublet regimen as first-line chemotherapy according to homologous recombination deficiency status and age group. **a** In the younger group, the median overall survival (OS) was significantly longer in the homologous recombination deficiency (HRD)-negative group compared to the HRD-positive group (22.0 months vs 13.6 months, *P* = 0.033). **b** In the older group, the median OS was similar between the HRD-negative and HRD-positive groups (30.5 months vs 33.3 months, *P* = 0.71). *OS* overall survival, *CI* confidence interval, *Mo* months, *No* number, *HRD* homologous recombination deficiency. **c** In the younger group, the median time to treatment failure (TTF) was significantly longer in the HRD-negative group than in the HRD-positive group (15.9 months vs 8.6 months, *P* = 0.041). **d** In the older group, the median TTF was comparable between the HRD-negative and HRD-positive groups (8.0 months vs 7.9 months, *P* = 0.68). *TTF* time to treatment failure, *CI* confidence interval, *Mo* months, *No* number, *HRD* homologous recombination deficiency

Finally, we evaluated the differential impact of HRD on OS and TTF in patients who received platinum-doublet regimens as first-line treatment, comparing younger and older groups. In the younger group, the median OS was significantly longer in HRD-negative patients compared to HRD-positive patients (22.0 months vs 13.6 months, *P* = 0.033) (Fig. 5a). In contrast, no significant difference in OS was observed between HRD-negative and HRD-positive cases in the older group (30.5 months vs 33.3 months, *P* = 0.71) (Fig. 5b). Similarly, in the younger group, the median TTF for platinum-doublet regimens was significantly longer in HRD-negative patients compared to HRD-positive patients (15.9 months vs 8.6 months, *P* = 0.041) (Fig. 5c). However, in the older group, no significant difference in TTF was found between HRD-negative and HRD-positive cases (8.0 months vs 7.9 months, *P* = 0.86) (Fig. 5d). These results indicate that HRD may be a predictive factor for the efficacy of platinum-doublet regimens, particularly in younger patients with gastric cancer.

## Discussion

In this study, we compared HRR gene variants between younger and older patients with GC to evaluate differences in genomic profiles and their associations with treatment efficacy and prognosis using a large-scale national genomic profiling database. Although the frequency of HRR gene variants was comparable between the two groups, no correlation was observed between HRR gene variants and high TMB or MSI-H status in the younger group. In contrast, HRR mutations were significantly associated with a high TMB and MSI-H status in the older group. Additionally, the impact of HRR variants on TTF and OS varied between the age groups.

Typically, HRD leads to elevated TMB and contributes to the MSI-H status [6, 13]. However, our findings revealed that this relationship holds true only in the older group and not in the younger cohort. A potential explanation is that the younger cohort with HRD exhibited fewer co-mutations, suggesting genomic stability (GS). In contrast, the older cohort with HRD often had co-mutations in genes such as *KRAS*, *APC*, *SMAD4*, and MMR, along with multiple HRR gene mutations, which may lead to a high TMB status. This aligns with previous reports indicating that a greater number of HRR gene mutations correlate with elevated TMB [14]. Additionally, the higher prevalence of smoking in the older cohort, a known risk factor for high TMB [15], may also contribute to these findings.

The association between HRD and sensitivity to platinum-based chemotherapy in gastric cancer, especially age-related differences in efficacy, is yet to be thoroughly investigated. In other malignancies, HRD has been associated with increased sensitivity to platinum-based chemotherapy [16–18]. Recent studies have suggested that gastric cancer patients with HRD show higher response rates to platinum-based therapies and longer progression-free survival than those without HRD [19]. However, our results demonstrate that the influence of HRD on TTF with platinum-based chemotherapy varies between younger and older patients. Several explanations for this are possible. The first explanation is the different frequencies of co-occurring genomic alterations and HRD between the two groups. Although HRD has been considered a detrimental prognostic factor [20–22], in younger patients, the lack of co-mutations and predominance of the GS subtype may undermine the favorable response typically associated with HRD. Conversely, in older patients, concurrent mutations in *APC* and *SMAD4*, along with multiple HRR gene mutations and high TMB or MSI-H status, may enhance chemotherapy sensitivity [23]. The second reason is that a higher overall proportion of patients received ICIs in the older group than in the younger group (Table 2). TMB was significantly higher in the older group, which may have contributed to their improved prognosis compared with that of the younger group (Fig. 4). This factor may have also contributed to the absence of differences in treatment efficacy and prognosis in the older group in contrast to the differences observed in the younger group according to HRD status.

Another intriguing aspect of this study was the varying association between HRD and cancer prognosis, depending on the age of onset, with different correlations observed in younger and older patients. This relationship may vary based on the type of variants in HRR genes [24], involved organs [5, 24] and tissue types [14]. Our study provides novel findings that the impact of HRD on OS differs between younger and older patients, mirroring its effect on TTF with platinum-based chemotherapy. Although the precise cause of this difference remains unclear, factors, such as histology, tumor location, and HRR gene variant types, may contribute to the varying effects of HRD on treatment efficacy and prognosis across age groups.

This study used a large-scale domestic genomic profiling cohort to reveal significant insights by examining both somatic and germline variants. The germline mutations were not evaluated in all cases because only the GenMineTOP and the NCC Oncopanel tests can detect germline variants. A germline-derived HRR gene mutation was identified in one older patient who underwent testing with the NCC Oncopanel. This case involved a small-scale *BRCA2* variant. Although this may be a limitation, it serves as an advantage by providing a broader understanding of the genetic landscape.

It is well-established that *Helicobacter pylori* infection is the primary risk factor for gastric cancer. However, due to the nature of the C-CAT database, which does not include infection status, we were unable to evaluate its contribution in this study. This remains an important limitation and an area for future investigation. To the best of our knowledge, this is the first study to explore how gene profile differences, including HRR genes, influence treatment efficacy and prognosis based on the timing of gastric cancer onset. Furthermore, our findings highlight that the effects of HRD on high TMB and MSI-H status vary depending on the timing of gastric cancer onset, which may be critical in determining treatment outcomes.

In conclusion, HRD status is differentially associated with molecular phenotypes and treatment response in younger versus older patients with gastric cancer. Our results suggest that HRR gene mutations, particularly in younger patients, may serve as potential biomarkers to guide therapeutic decisions. Given the limited effectiveness of standard platinum-based chemotherapy in HRD-positive younger patients, our findings underscore the need for age-specific precision oncology approaches, including the development of novel HRD-targeted therapies.

## Abbreviations

C-CAT: Center for Cancer Genomics and Advanced Therapeutics
CGP: Comprehensive genomic profiling
CI: Confidence interval
CNV: Copy number variants
ECOG PS: Eastern Cooperative Oncology Group Performance Status
HER2: Human epidermal growth factor receptor 2
HRD: Homologous recombination deficiency
HRR: Homologous recombination repair
ICI: Immune checkpoint inhibitor
MSI: Microsatellite instability
OS: Overall survival
QC: Quality check
SNV: Single-nucleotide variants
TMB: Tumor mutation burden
TTF: Time to treatment failure
VUS: Variant of unknown significance
